# Glycemic Control With Layperson-Delivered Telephone Calls vs Usual Care for Patients With Diabetes: A Randomized Clinical Trial

**DOI:** 10.1001/jamanetworkopen.2024.48809

**Published:** 2024-12-10

**Authors:** Maninder K. Kahlon, Nazan S. Aksan, Rhonda Aubrey, Nicole Clark, Maria Cowley-Morillo, Carolina DuBois, Carlos Garcia, Julia Guerra, David Pereira, Mathew Sither, Steven Tomlinson, Sandy Valenzuela, M. Renee Valdez

**Affiliations:** 1 Department of Population Health, Dell Medical School, The University of Texas at Austin; 2 Seminary of the Southwest, Austin, Texas; 3 Lone Star Circle of Care, Georgetown, Texas

## Abstract

**Importance:**

Diabetes is associated with emotional distress and poor mental health, especially for individuals with low income, hindering patients’ ability to manage their condition. The health care system’s workforce constraints limit its capacity to holistically support patients.

**Objective:**

To assess the effectiveness of layperson-delivered empathetic engagement over the telephone in helping improve glycemic management for patients with diabetes.

**Design, Setting, and Participants:**

This parallel-arm randomized clinical trial with blinded outcome assessment was conducted from February 12, 2022, to April 15, 2023, with final measurements on November 18, 2023, among 260 patients with uncontrolled diabetes from a federally qualified health center in Austin, Texas, engaging telephonically from home.

**Intervention:**

Patients assigned to the intervention group received empathy-oriented telephone calls by community-hired laypeople for 6 months, while those assigned to the control group received usual care. Patients were stratified by baseline score (≥5 vs <5) on the depressive symptom scale of the 9-item Patient Health Questionnaire (PHQ-9).

**Main Outcomes and Measures:**

The primary outcome was hemoglobin A_1c_ level at baseline, 3 months, and 6 months, assessed for interaction between time and trial arm. Secondary outcomes were self-perceptions of managing diabetes, diabetes-related behaviors and distress, and mental health symptoms (measured via surveys). Analysis was performed on an intention-to-treat basis.

**Results:**

Of 260 participants (mean [SD] age, 49.5 [10.1] years; 163 of 259 women [62.9%]; 176 of 203 [86.7%] with annual income <$40 000) enrolled, 6 withdrew. At 6 months, 204 of 254 (80.3%; intervention, 109 of 127 [85.8%] and control, 95 of 127 [74.8%]) returned for measurements. Participants in the intervention group had statistically significant mean (SD) decreases in hemoglobin A_1c_ level at 6 months (from 10.0% [1.9%] to 9.3% [2.0%]) compared with those in the control group (from 9.8% [1.6%] to 9.7% [2.3%]) (*P* = .004). The within-person change in hemoglobin A_1c_ level was −0.7% (95% CI, −1.0% to −0.4%) for the intervention group and 0.02% (95% CI, −0.4% to 0.4%) for the control group. For the subgroup with a PHQ-9 score of 5 or more at baseline (38.1% [99 of 260]), the within-person change in hemoglobin A_1c_ was −1.1% (95% CI, −1.8% to −0.5%) for the intervention group and 0.1% (95% CI, −0.7% to 0.8%; *P* = .004) for the control group. For the subgroup with a PHQ-9 score less than 5, the within-person change in hemoglobin A_1c_ was −0.4% (95% CI, −0.8% to −0.1%) for the intervention group and −0.02% (95% CI, −0.5% to 0.5%; *P* = .21) for the control group. At 6 months, 91.7% of the participants (99 of 108) responded that the program was very or extremely beneficial.

**Conclusions and Relevance:**

In this randomized clinical trial of telephone-based layperson-delivered empathetic engagement, patients with diabetes and low income achieved clinically meaningful improvements in glycemic control. With workforce constraints, layperson-delivered programs for diabetes show promise.

**Trial Registration:**

ClinicalTrials.gov Identifier: NCT05173675

## Key Points

**Question:**

Can layperson-delivered telephone engagement affect glycemic control for patients with diabetes?

**Findings:**

This randomized clinical trial of 260 patients with uncontrolled diabetes at a federally qualified health center showed statistically significant improvements in hemoglobin A_1c_ level of −0.7% for those who received empathy-oriented telephone calls for 6 months vs 0.02% for control patients who received usual care. For the subgroup of patients with mild or greater depressive symptoms at baseline, statistically significant improvements in hemoglobin A_1c_ level were greater, at −1.1% for the intervention arm vs 0.1% for controls.

**Meaning:**

This study suggests that layperson-delivered, empathy-focused telephone engagement can improve glycemic control for patients with diabetes.

## Teaser

This randomized clinical trial assesses the effectiveness of layperson-delivered empathetic engagement over the telephone in helping improve glycemic management for patients with diabetes.

## Introduction

To better manage diabetes, lifestyle change is necessary. However, the challenges of day-to-day life with diabetes generates distress and reduces self-confidence in the ability to manage the condition and implement behavioral change.^1,2,3^ Diabetes is also associated with mental health conditions, especially depression,^4,5,6^ even when depression is subclinical,^7^ which creates further barriers to behavior change.^8^ These diabetes-specific as well as broader mental health challenges faced by patients with diabetes are being recognized, but limited health care touchpoints constrain addressing them. Support via behavioral health, when available, is offered only for those who reach a clinical threshold, with an insufficient workforce severely limiting capacity,^9^ particularly for patients with low socioeconomic status^10^ who lack resources.^11,12^

To support the emotional and mental health needs of patients with chronic health conditions such as diabetes, alternate workforce models with nonmedical laypeople are being assessed.^13,14,15^ Approaches used include cognitive behavioral therapy or motivational interviewing, with training lasting from days to months.^16,17,18^ Nonmedical laypeople also play other roles with or without the mental health training, including navigation and education,^19^ and can spend the time to focus on the needs of patients.^13^ Assessing the extent to which more laypeople in the workforce can be useful can help us scope an optimal role for them. With the prevalence of diabetes only increasing and no improvement in its control,^20^ the unmet need is significant, especially for those with lower socioeconomic status.^21,22,23,24^

We wished to assess whether empathetic conversations provided over the telephone by a layperson without a health care background could benefit patients with uncontrolled diabetes. In a previous study, laypeople were trained on a telephone-based empathetic connection protocol that prioritized participant interests, with improvements in depressive symptoms relative to controls demonstrated in 4 weeks.^25,26^ We extended the duration of this program and customized it to support patients with uncontrolled diabetes and low income, and we report on the prespecified primary outcome of glycemic management relative to usual care. We hypothesized that sharing the day-to-day aspects of living with diabetes with an empathetic listener could reduce emotional burden, lower barriers to lifestyle changes, and improve disease management. Because depressive symptoms are associated with glycemic control, we balanced depressive risk in the trial arms. We also explored measures of patients’ self-perception of managing diabetes and related behaviors and distress, as well as mental health.

## Methods

### Design

We conducted a parallel-arm superiority trial with 1:1 allocation via stratified and blocked randomization. Participants were recruited from a federally qualified health center in central Texas and randomly assigned, on a rolling basis, to receive the 6-month program or continue with usual care, from February 12, 2022, to April 15, 2023, with final measurements on November 18, 2023. Patients received biomedical and survey-based measurements at clinic sites at baseline, 3 months, and 6 months, for which they were compensated and provided written informed consent. This trial was approved by the institutional review board at the University of Texas at Austin and follows the Consolidated Standards of Reporting Trials (CONSORT) reporting guideline (trial protocol in Supplement 1).

### Participants

Participants were recruited to talk about living with diabetes and the demands of controlling blood glucose. The clinic texted information about the opportunity to eligible patients. Those interested learned more on the telephone and signed a consent form at the first baseline measurement visit.

Persons 21 years of age or older were eligible for inclusion if they had at least 1 clinic appointment and 1 hemoglobin A_1c_ (HbA_1c_) measurement of 8.0% or greater in the prior 12 months, with an HbA_1c_ level of 7.5% at baseline measurement. Exclusion criteria included cognitive impairment, pregnancy, cancer, and end-stage kidney disease. Participants also had to complete the 9-item Patient Health Questionnaire (PHQ-9)^27^ at baseline for stratified randomization. To balance mental health in both arms, we chose the well-studied PHQ-9 but with a cutoff score of 5 to include subclinical depressive symptoms and broader emotional challenges.

### Randomization

After baseline data collection, participants were stratified by baseline score on the depressive symptom scale of the PHQ-9 (≥5 and <5) and randomized, 1:1 in blocks of 2 and 4, into intervention and usual care arms. Randomized allocation was generated by the biostatistician (N.S.A.) and uploaded to REDCap.^28^ A manager (R.A. and S.V.) assigned participants in the intervention group sequentially to a caller, up to 15 participants each, adjusting as necessary for language preference (Spanish or English). Participants in the usual care group were notified by text message of their assignment and measurement dates.

### Intervention

Participants received telephone calls of unlimited duration from a dedicated caller for 6 months to talk about their life with diabetes. Callers asked questions, without scripts, about whatever participants raised themselves (eg, their neighbor, dinner, or frustrations with managing blood glucose). Eleven callers were hired for telephone-based hourly work, without requiring health care experience but interviewed for empathetic orientation, including curiosity and ability to shift perspective. Most callers were bilingual (English and Spanish), with bachelor’s degrees, and from diverse professions, such as a recent mother, a musician, and a retired teacher. Callers received 8 hours of training,^29^ including conversational practice with scenarios specific to life with diabetes for patients with low income, basic diabetes education (using publicly available information),^29^ the program protocol including prioritizing participant preferences, and telephone and desktop-based calling software (caller tool). Program fidelity was reviewed through virtual biweekly group meetings and through data collected from the caller tool.

Participants received 3 telephone calls in the first week from their assigned caller and then chose 1 to 5 calls per week for the next 3 weeks and subsequently 1 call every 1 or 2 weeks. At the end of the program, callers used guidelines to “graduate” participants off the program. In the first week of the program, callers offered participants the option to receive a pedometer or smart scale (<$25). During the program, callers also compiled and mailed 2 letters to their panels that abstracted anonymized lessons learned from participants. Participants were also mailed 2 gifts (<$25 each) selected by callers that reflected their understanding of participants’ preferences (eg, a yoga mat or a cookbook).

### Measurement

Participants were scheduled for 3-month and 6-month measurements at clinic sites, with a maximum of 10 attempts if unreachable. Trained study staff, blinded to arm, took measurements from 2 weeks before the end of the 13th and 26th weeks, respectively, of their baseline measurements. Three-month measures occurred a mean (SD) of 13.7 (1.9) weeks after baseline measurements, and 6-month measures occurred a mean (SD) of 26.4 (2.3) weeks after baseline measurements. Hemoglobin A_1c_ was measured with finger stick on a portable device. Demographic characteristics, including race and ethnicity, were collected via self-reported surveys at baseline on a tablet. The source of racial and ethnic categories was the US Office of Management and Budget; race and ethnicity were collected in this study to support future scaling. Mental health measures included symptoms of depression (PHQ-9; α = 0.91)^27^ and anxiety (7-item Generalized Anxiety Disorder scale [GAD-7]; α = 0.93).^30^ Measures of self-perception of ability to manage diabetes, related behaviors, and distress included (1) their confidence in conducting diabetes-improving behaviors, such as choosing appropriate foods, exercising, or managing blood glucose levels (Stanford Self-Efficacy for Diabetes Scale [SEDS]; α = 0.88)^31^; (2) their confidence in managing their diabetes broadly, such as feeling as if they can meet their goals and handle themselves well, and feeling effective in changing behaviors (Perceived Diabetes Self-Management Scale [PDSMS]; α = 0.76)^32^; (3) feeling overwhelmed by the demands of living with diabetes or feeling like they are failing on diabetes routines (2-item Diabetes Distress Screening Scale [DDSS]; α = 0.84)^33^; and (4) how they deviate from medication instructions (Medication Adherence Report Scale [MARS; α = 0.78).^34^ In choosing survey tools, we prioritized access to Spanish versions and low participant burden. Higher scores on the SEDS,^35^ PDSMS,^32^ and MARS^34^ indicate better emotional health and adherence, while higher scores on the DDSS^33^ indicate higher levels of distress. Program satisfaction questions were also assessed at 6 months.

### Statistical Analysis

We used mixed linear models with maximum likelihood with random person intercepts in an intention-to-treat framework for the analysis of primary and secondary outcomes. These methods permit reliance on all available data at all time points irrespective of whether participants missed a measurement, compared with traditional mixed-design analysis of variance models that demand listwise deletion.^36^ Time was modeled categorically as target-months (ie, 0 for baseline [reference], 3-month, and 6-month assessments). The models included main effects of group (reference, control), stratifier (reference, PHQ-9 score <5 at baseline), and their 2-way interactions with time. Clustering within callers was tested and removed if the intraclass correlation was negligible or null. The total number of days elapsed from baseline to final assessment was modeled as a person-level covariate. The target term of interest was the interaction of group with time at 6 months. We examined the gains in HbA_1c_ level with total dose measures (number of successful calls and total number of minutes) using Pearson correlations in which control participants were assigned zero calls and zero minutes.

For secondary outcomes (PHQ-9, GAD-7, SEDS, PDSMS, DDSS, and MARS), to guard against inflation of the type I error rate, we relied on confirmatory factor analysis (CFA) methods to inform formation of composite scores.^37^ Baseline measures of all 6 scale scores (prior to intervention) were submitted to a CFA with maximum likelihood estimation. The models tested single-factor and 2-factor (separating mental health [MH model; measured by the PHQ-9 and GAD-7] from self-perceptions of ability to manage diabetes, related behaviors and distress [measured by the SEDS, PSDMS, DDSS, and MARS], which we term *diabetes-related emotional health* [DMEH model]) vs 3-factor models (further separating medication adherence [measured by the MARS] with less affective item content, from DMEH). The models were compared with nested log likelihood χ^2^ tests. Guided by the results of the CFA, we formed unit-weighted composite measures that aligned with the best-fitting factor structure. These composites were then submitted to similar linear mixed-effect models as the primary outcome. The α level for the primary and composite secondary outcomes was set at .05 with 2-sided *P* values. Analyses of dropout patterns across groups used χ^2^ independence tests, with α also set at 0.05 with 2-sided *P* values. All data management and analyses were completed in Stata, version 16.0 (StataCorp LLC),^38^ except Mplus, version 8.0 (Muthén & Muthén)^39^ for CFA analyses.

## Results

### Demographics

The sample included 260 participants (mean [SD] age, 49.5 [10.1 years]; 163 of 259 women [62.9%] and 96 of 259 men [37.1%]; 21 of 221 African American [9.5%], 6 of 221 Asian [2.7%], 1 of 221 Hawaii or Other Pacific Islander [0.5%], 197 of 251 Hispanic or Latino [78.5%], 14 of 221 Native American or Alaska Native [6.3%], 109 of 221 White [49.3%], and 64 of 221 of other race or ethnicity [29.0%]; 176 of 203 [86.7%] with income below $40 000; and 176 of 233 [75.5%] with a high school degree or less) (Table 1). From February 2022 to April 2023 (Figure), we received interest via response to the clinic’s text message from 1077 eligible patients, of whom we were able to contact 597. Of these, 260 were randomized to the intervention group (n = 129) and the usual care control group (n = 131). Subsequently, at 3 months, 4 patients withdrew (2 from the intervention group and 2 from the usual care group), and 213 of 256 (83.2%; intervention, 111 of 127 [87.4%] and control, 102 of 129 [79.1%]) returned for measurement visits, and at 6 months, 2 patients withdrew from the usual care group, and 204 of 254 (80.3%; intervention, 109 of 127 [85.8%] and control, 95 of 127 [74.8%]) returned for measurement visits. Participants in the usual care group were more likely to miss assessments relative to those in the intervention group (χ^2^_1_ = 4.1; *P* = .045). There was no evidence that dropout patterns were related to status of baseline stratifier (χ^2^_1_ <1; *P* = .58).

**Table 1.** Demographic Characteristics of the Sample

Variable	Whole sample (N = 260)		Control (n = 131)		Intervention (n = 129)	
	No. (%)	Value	No. (%)	Value	No. (%)	Value
Age, mean (SD), y						
At diagnosis	251 (96.5)	36.3 (12.2)	126 (96.2)	36.5 (11.4)	125 (96.9)	36.1 (10.1)
At time of enrollment	260 (100)	49.5 (10.1)	131 (100)	49.4 (9.6)	129 (100)	49.7 (10.5)
Gender, No. (%)						
Female	259 (99.6)	163 (62.9)	131 (100)	85 (64.9)	128 (99.2)	78 (60.9)
Male	259 (99.6)	96 (37.1)	131 (100)	46 (35.1)	128 (99.2)	50 (39.1)
Race and ethnicity, No. (%)						
African American	221 (85.0)	21 (9.5)	115 (87.8)	10 (8.7)	106 (82.2)	11 (10.4)
Asian	221 (85.0)	6 (2.7)	115 (87.8)	4 (3.5)	106 (82.2)	2 (1.9)
Hawaii or Other Pacific Islander	221 (85.0)	1 (0.5)	115 (87.8)	1 (0.9)	106 (82.2)	0
Hispanic or Latino	251 (96.5)	197 (78.5)	126 (96.2)	104 (82.5)	125 (96.9)	93 (74.4)
Native American or Alaska Native	221 (85.0)	14 (6.3)	115 (87.8)	7 (6.1)	106 (82.2)	7 (6.6)
White	221 (85.0)	109 (49.3)	115 (87.8)	62 (53.9)	106 (82.2)	47 (44.3)
Other^a^	221 (85.0)	64 (29.0)	115 (87.8)	32 (27.8)	106 (82.2)	32 (30.2)
High school education or less, No. (%)	233 (89.6)	176 (75.5)	119 (90.8)	89 (74.8)	114 (88.4)	87 (76.3)
Household income, No. (%)						
<$10 000	203 (78.1)	60 (29.6)	105 (80.2)	33 (31.4)	98 (76.0)	27 (27.6)
$10 000-$19 999	203 (78.1)	63 (31.0)	105 (80.2)	34 (32.4)	98 (76.0)	29 (29.6)
$20 000-$39 999	203 (78.1)	53 (26.1)	105 (80.2)	25 (23.8)	98 (76.0)	28 (28.6)
$40 000-$59 999	203 (78.1)	17 (8.4)	105 (80.2)	7 (6.7)	98 (76.0)	10 (10.2)
$60 000-$80 000	203 (78.1)	6 (3.0)	105 (80.2)	5 (3.8)	98 (76.0)	1 (1.0)
>$80 000	203 (78.1)	4 (2.0)	105 (80.2)	1 (1.0)	98 (76.0)	3 (3.1)
Married or living with partner, No. (%)	249 (95.8)	128 (51.4)	125 (95.4)	70 (56.0)	124 (96.1)	58 (46.8)
No insurance, No. (%)	247 (95.0)	89 (36.0)	124 (94.7)	49 (39.5)	123 (95.3)	46 (37.4)
Type of insurance, No. (%)^b^						
Medicare or Medicaid	156 (60.0)	39 (25.0)	76 (58.0)	21 (28.0)	80 (62.0)	18 (22.5)
Employer based	156 (60.0)	17 (10.9)	76 (58.0)	9 (12.0)	80 (62.0)	8 (10.0)
MAP^c^	156 (60.0)	80 (51.3)	76 (58.0)	36 (47.3)	80 (62.0)	44 (55.0)
Other^d^	156 (60.0)	20 (12.8)	76 (58.0)	10 (13.2)	80 (62.0)	10 (12.5)
Other comorbidities, No. (%)	260 (100)	166 (63.8)	131 (100)	82 (62.6)	129 (100)	84 (65.1)
At least 1 SDOH, No. (%)	260 (100)	142 (54.6)	131 (100)	56 (42.7)	129 (100)	42 (32.6)

Abbreviations: MAP, Medical Access Program (local tax-funded health benefits for uninsured individuals); SDOH, social determinant of health.

^a^ Participants who chose “other” in response to race and ethnicity options were not given any specific alternatives, simply “some other race.”

^b^ Percentage is reported over a denominator with any type of insurance.

^c^ County health for uninsured.

^d^ Self-report as Patient Protection and Affordable Care Act or exchange, federally qualified health center–supported care.

**Figure.**
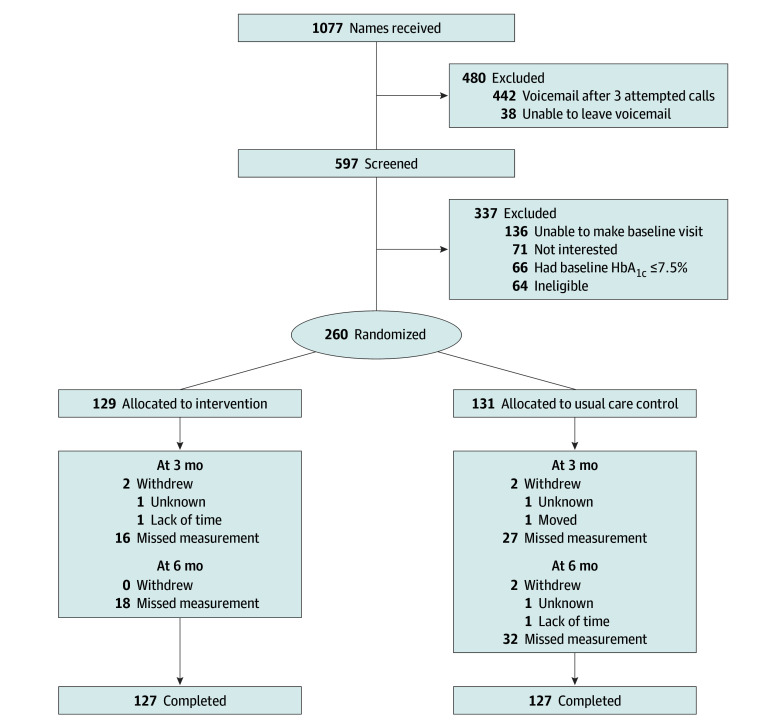
Flow Diagram HbA_1c_ indicates hemoglobin A_1c_.

### Calls

A mean of 20.0 (6.6) calls were completed per participant in the intervention group (range, 7-36 calls; 95% CI, 19.1-21.6 calls). The mean (SD) total call duration per participant was 360.6 (379.0) minutes (range, 32-1814 minutes; 95% CI, 289.0-432.2 minutes).

### Primary Outcome

The intraclass correlation associated with callers was negligible (9.78 × 10^−14^) and thus removed when estimating mixed linear models. Table 2 shows descriptive data for the primary outcome, HbA_1c_ level, at baseline, 3-month, and 6-month assessments both combined and disaggregated by PHQ-9 score stratifier status at baseline (eTables 1 and 2 in Supplement 2 show outcomes by gender). The mean (SD) HbA_1c_ level for the intervention group decreased from 10.0% (1.9%) at baseline to 9.3% (2.0%) at 6 months, whereas the mean (SD) HbA_1c_ level for the control group decreased from 9.8% (1.6%) at baseline to 9.7% (2.3%) at 6 months (*P* = .004 for time-by-group interaction at 6 months but not significant at 3 months). The within-person change in HbA_1c_ level at 6 months relative to baseline was −0.7% (95% CI, −1.0% to −0.4%) for the intervention group and 0.02% (95% CI, −0.4% to 0.4%) for the control group. For participants with a PHQ-9 score of 5 or more at baseline (38.1% [99 of 260]), the mean (SD) HbA_1c_ level in the intervention group (38.0% [49 of 129]) decreased from 10.1% (1.9%) at baseline to 8.9% (1.8%) at 6 months, whereas the mean (SD) HbA_1c_ level in the control group (38.2% [50 of 131]) increased from 10.1% (1.7%) at baseline to 10.2% (2.7%) at 6 months (*P* = .004). For this subgroup, the mean within-person change was −1.1% (95% CI, −1.8% to −0.5%) for the intervention group and 0.1% (95% CI, −0.7% to 0.8%) for the control group. For the subgroup with a PHQ score less than 5, the within-person change in HbA_1c_ level was −0.4% (95% CI, −0.8% to −0.1%) for the intervention group and −0.02% (95% CI, −0.5% to 0.5%) for the control group. The improvements in HbA_1c_ level from baseline to 6-month assessments for the full sample were correlated with the total number of calls (*r*[195 participants] = −0.17; *P* = .02) and total number of minutes (*r*[195 participants] = −0.14; *P* = .048), with controls assigned a value of zero.

**Table 2.** Hemoglobin A_1c_ Levels and Within-Person Change Over Time

Group	Mean (SD) hemoglobin A_1c_, % [No.]			Change (from baseline to 6 mo)		*P* value
	At baseline	At 3 mo	At 6 mo	Mean (SD), % [No.]	95% CI, %	
Usual care combined	9.8 (1.6) [130^a^]	9.7 (1.9) [101]	9.7 (2.3) [95]	0.02 (2.0) [95]	−0.4 to 0.4	.004
Intervention combined	10.0 (1.9) [129]	9.7 (2.1) [110]	9.3 (2.0) [108^b^]	−0.7 (1.6) [108]	−1.0 to −0.4	
Usual care PHQ-9 score <5^c^	9.7 (1.6) [80]	9.6 (1.7) [60]	9.4 (1.9) [59]	−0.02 (1.8) [59]	−0.5 to 0.5	.21
Intervention PHQ-9 score <5^c^	9.9 (1.9) [80]	9.9 (2.2) [68]	9.6 (2.1) [69]	−0.4 (1.4) [69]	−0.8 to −0.1	
Usual care PHQ-9 score ≥5^c^	10.1 (1.7) [50]	10.0 (2.2) [41]	10.2 (2.7) [36]	0.1 (2.3) [36]	−0.7 to 0.8	.004
Intervention PHQ-9 score ≥5^c^	10.1 (1.9) [49]	9.4 (2.0) [42]	8.9 (1.8) [39]	−1.1 (2.0) [39]	−1.8 to –0.5	

Abbreviation: PHQ-9, 9-item Patient Health Questionnaire.

^a^ Of 131 patients in the usual care group that came in for measurement at baseline, 1 hemoglobin A_1c_ value was lost.

^b^ Of 109 patients in the intervention group that came in for measurement at 6 months, 1 hemoglobin A_1c_ value was lost.

^c^ Stratifier based on PHQ-9 total score at baseline being less than 5 or greater than or equal to 5.

### Secondary Outcomes

To explore trends in PHQ-9, GAD-7, SEDS, PDSMS, DDSS, and MARS scores without boosting type I error rates, we relied on CFA to inform how to pool these measures into composite scores as described in Methods. Mplus^39^ results showed that the 2-factor model MH (GAD-7, PHQ-9) and DMEH (SEDS, PDSMS, DDSS, and MARS), was the best fitting model, improving fit over the single-factor model (change in χ^2^_2_ = 132.9; *P* < .001). There was no evidence that separating MARS from DMEH improved model fit (change in χ^2^_1_ = 2.34; *P* = .13).

We formed unit-weighted composite scores for the 2-factor solution (MH and DMEH) by rescaling the total scores to range between 0 and 1 and then averaging the rescaled totals. The descriptive data for these 2 composite scores are shown in Table 3 below the individual scales included in each composite. For the MH model, higher composite scores reflect worsened mental health, while for the DMEH model, higher composite scores reflect improved emotional health. The intraclass correlation for the clustering effect of callers was negligible for the MH model (1.88 × 10^−28^) and the DMEH model (2.45 × 10^−24^) and thus removed in estimating linear mixed-effect regressions. The composite score for DMEH for those who had a PHQ-9 score of 5 or more at baseline showed statistically significant change at 6 months (difference from baseline, 0.09 [95% CI, 0.05-0.13] for intervention group and 0.04 [95% CI, –0.01 to 0.09] for control group; *P* = .03 for time × group interaction), but the composite MH score did not. When individual scales included in these composite scores were also submitted to similar analyses, the individual effects were statistically null.

**Table 3.** Descriptive Statistics for Composite Measures of MH and DMEH Across Time

Outcome and group^a^	Mean (SD) [No.]			Change (from baseline to 6 mo)		***P* value**
**At baseline**	**At 3 mo**	**At 6 mo**	**Mean (SD) [No.]**	**95% CI**
**PHQ-9 score** ^b^						
<5						
Usual care	1.67 (1.52) [81]	1.73 (2.19) [56]	1.65 (2.0) [55]	0.11 (1.92) [55]	−0.41 to 0.63	.35
Intervention	1.49 (1.3) [80]	1.4 (1.74) [63]	1.65 (3.51) [70]	0.54 (3.65) [70]	−0.34 to 1.41	
≥5						
Usual care	10.92 (5.23) [50]	8.79 (6.09) [38]	8.78 (5.34) [36]	−1.61 (5.04) [36]	−3.32 to 0.09	.48
Intervention	9.98 (5.15) [49]	8.07 (5.72) [41]	7.72 (4.94) [39]	−2.54 (4.17) [39]	−3.89 to −1.19	
**GAD-7 score**						
<5						
Usual care	2.68 (3.64) [81]	2.2 (2.88) [60]	2.2 (2.98) [59]	−0.46 (2.70) [59]	−1.16 to 0.25	.14
Intervention	1.74 (2.45) [80]	1.94 (2.72) [68]	1.24 (0.93) [70]	−0.33 (1.98) [70]	−0.80 to 0.14	
≥5						
Usual care	9.04 (5.24) [50]	9 (6.74) [41]	7.36 (4.96) [36]	−1.31 (0.65) [36]	−2.62 to 0.01	.16
Intervention	8.9 (5.73) [49]	8.29 (6.24) [42]	9.03 (5.72) [39]	0.46 (5.04) [39]	−1.17 to 2.09	
**MH composite score**						
<5						
Usual care	0.09 (0.1) [81]	0.09 (0.09) [56]	0.09 (0.09) [55]	−0.01 (0.08) [55]	−0.03 to 0.02	.63
Intervention	0.06 (0.07) [80]	0.07 (0.09) [63]	0.06 (0.13) [70]	0.01 (0.09) [70]	−0.02 to 0.02	
≥5						
Usual care	0.42 (0.19) [50]	0.37 (0.24) [38]	0.34 (0.2) [36]	−0.06 (0.16) [36]	−0.11 to −0.01	.53
Intervention	0.40 (0.21) [49]	0.35 (0.24) [41]	0.36 (0.21) [39]	−0.04 (0.17) [39]	−0.09 to 0.02	
**PDSMS score**						
<5						
Usual care	24.02 (5.92) [81]	26.65 (6.04) [60]	26.56 (5.83) [59]	1.93 (7.01) [59]	0.11 to 3.76	.66
Intervention	25.05 (5.89) [80]	26.1 (6.48) [68]	27.83 (5.48) [70]	2.64 (5.54) [70]	1.32 to 3.96	
≥5						
Usual care	21.16 (5.02) [50]	22.2 (6.04) [41]	22.83 (6.17) [36]	1.61 (7.35) [36]	−0.88 to 4.10	.08
Intervention	20.73 (5.96) [49]	23.93 (6.19) [42]	24.87 (6.74) [39]	3.95 (8.53) [39]	1.18 to 6.71	
**DDSS score**						
<5						
Usual care	7.37 (2.87) [81]	8.02 (2.84) [60]	8.27 (2.84) [59]	1.20 (2.74) [59]	0.49 to 1.92	.87
Intervention	7.69 (2.97) [80]	8.49 (2.62) [68]	8.76 (2.54) [70]	1.23 (2.61) [70]	0.61 to 1.85	
≥5						
Usual care	5.68 (2.85) [50]	5.37 (2.95) [41]	6.22 (3.13) [36]	0.47 (3.19) [36]	−0.61 to 1.55	.15
Intervention	5.31 (2.79) [49]	6.12 (3.09) [42]	6.79 (2.87) [39]	1.33 (2.73) [39]	0.45 to 2.22	
**SEDS score**						
<5						
Usual care	49.44 (18.92) [81]	51.77 (17.78) [60]	53.54 (19.07) [59]	3.47 (16.52) [59]	−0.83 to 7.78	.72
Intervention	52.08 (19.09) [80]	53.43 (20.27) [68]	57.11 (19.11) [70]	5.44 (18.69) [70]	0.99 to 9.90	
≥5						
Usual care	35 (15.45) [50]	41.95 (15.84) [41]	39.5 (17.88) [36]	5.39 (15.87) [36]	0.02 to 10.75	.33
Intervention	38.67 (13.21) [49]	46.81 (17.03) [42]	47.03 (18.43) [39]	8.44 (20.08) [39]	1.93 to 14.95	
**MARS score**						
<5						
Usual care	21.44 (3.66) [81]	22.05 (3.61) [60]	21.42 (4.67) [59]	0.20 (4.76) [59]	−1.04 to 1.44	.87
Intervention	21.59 (3.41) [80]	21.32 (3.39) [68]	21.74 (3.7) [70]	0.04 (3.42) [70]	−0.77 to 0.86	
≥5						
Usual care	19.16 (4.65) [50]	19.61 (4.48) [41]	19.14 (4.28) [36]	0.28 (4.14) [36]	−1.12 to 1.68	.12
Intervention	17.96 (4.01) [49]	19.5 (4.57) [42]	19.62 (3.72) [39]	1.23 (3.58) [39]	0.07 to 2.39	
**DMEH composite score**						
<5						
Usual care	0.68 (0.13) [81]	0.72 (0.14) [60]	0.72 (0.13) [59]	0.05 (0.13) [59]	0.02 to 0.09	.71
Intervention	0.70 (0.14) [80]	0.72 (0.14) [68]	0.75 (0.13) [70]	0.06 (0.10) [70]	0.04 to 0.08	
≥5						
Usual care	0.55 (0.12) [50]	0.58 (0.16) [41]	0.59 (0.16) [36]	0.04 (0.14) [36]	−0.01 to 0.09	.03
Intervention	0.54 (0.12) [49]	0.62 (0.15) [42]	0.64 (0.15) [39]	0.09 (0.13) [39]	0.05 to 0.13	

Abbreviations: DDSS, Diabetes Distress Screening Scale; DMEH, diabetes-related emotional health; GAD-7, 7-item Generalized Anxiety Disorder Scale; MARS, Medication Adherence Report Scale; MH, mental health; PDSMS, Perceived Diabetes Self-Management Scale; PHQ-9, 9-Item Patient Health Questionnaire; SEDS, Stanford Self-Efficacy for Diabetes Scale.

^a^ High scores on the PDSMS, DDSS, MARS, and SEDS reflect better diabetes management, less distress, better adherence, and higher efficacy; high values in the DMEH composite score reflect better diabetes-related emotional health. High scores on the PHQ-9 and GAD-7 and the MH composite score reflect greater symptoms of depression or anxiety.

^b^ Stratifier based on PHQ-9 total score at baseline being less than 5 or greater than or equal to 5.

### Program Satisfaction

At 6 months, 108 of 108 patients (100%) responded to the question, “how beneficial was the program to you?”, with 0 of 108 choosing “not at all,” 0 of 108 choosing “slightly,” 9 of 108 (8.3%) choosing “moderately,” 59 of 108 (54.6%) choosing “very,” and 40 of 108 (37.0%) choosing “extremely.” In response to the question, “how likely are you to recommend the program to a friend or family member?”, the mean (SD) score from 1 (not likely) to 10 (extremely likely) was 8.7 (2.3), with 63 of 98 (64.3%) selecting 10. With 101 of 108 patients (93.5%) responding, qualitative responses to the question “What did you find most useful in talking to your dedicated partner?” included comments on motivational support (“she helped me a lot with encouragement”), emotional support (eg, “felt better talking”), informal instrumental support (eg, “the recipes she gave me”), and valuing the caller’s interest in them (“the compassion and consistency”).

## Discussion

Patients with low income and uncontrolled diabetes spoke on the telephone with empathetic laypeople for 6 months and experienced statistically significant and clinically meaningful glycemic control (HbA_1c_ level, >0.5%^40^) relative to usual care controls, with a small dose-response association between call frequency or duration and improvement in HbA_1c_ level. Patients with baseline depressive risk (PHQ-9 score ≥5) showed over 1% improvement, associated with reduced diabetes-related complications.^41^ This subset also showed evidence of improved DMEH, including self-perception of ability to manage diabetes, related behaviors, and distress.

Psychological interventions used to support diabetes management, delivered by clinicians and paraprofessionals, include cognitive behavioral therapy, motivational interviewing, and collaborative care integrating these or other behavioral health methods with primary care. Randomized clinical trials have shown mixed or small effects of these interventions on glycemic management, with meta-analyses estimating mean improvements in HbA_1c_ level between −0.28% and −0.34%,^42,43,44^ whereas interventions implemented for patients with depression comorbid with diabetes have generally improved depression, with heterogeneous effects on glycemic management.^45,46,47,48^ In this trial, we saw improvements in HbA_1c_ levels relative to usual care on the higher end of improvements previously seen, with mean per-person improvements of −0.7% and improvements for those with depressive risk of −1.1%.

Our approach differed from existing health care models, including those that benefit from other paraprofessional roles, such as community health workers, in its goals and mechanics.^19,49,50^ The goal was not to accomplish tasks but to converse to learn more about the patient. Patients chose the frequency and length of calls and discussed their own interests. When they expressed needs, which they were more likely to do because they were being called regularly by someone they trusted, patients were supported in connecting back to the health system. This reversal of focus, starting with the patients on their own terms, may have increased their sense of autonomy,^51,52,53^ enabling healthy lifestyle changes aligned with personal preferences.

Although glycemic control improved, our exploratory analysis of mental health and diabetes-related emotional distress, including self-perceptions of diabetes management and behaviors, found evidence of improvement in diabetes-related emotional distress only for those with depressive risk at baseline. This subpopulation also showed the greatest improvement in glycemic control. Although both depression and diabetes-related distress are associated with poor glycemic control, the conceptual relationships remain unclear.^54^ Some studies suggest that diabetes-related distress influences glycemic control more than depression^55,56,57^ and that the effects of depression are mediated by diabetes distress.^58,59^ In turn, improving depression is not always associated with improvements in glycemic management.^45,47^ Our exploratory results support the importance of addressing patients’ diabetes-related emotional distress to effectively improve glycemic control.

### Limitations

This study has some limitations. A larger sample size might have allowed us to identify evidence for mental health improvements with smaller effect sizes. In our choices of scales for assessing distress, we were able to assess perceptions of behavior change, but we did not measure objectively physical activity or diet. Differential dropout in the usual care arm (75% follow-up) at 6 months was greater than for the intervention arm (86% follow-up). Although this is a limitation for precision of estimates, it would be of greater concern had it been present in the intervention arm. Finally, it would be useful to understand the benefits of a longer program duration, or if effects are sustained after the program ends.

## Conclusions

This randomized clinical trial found that a focus on consistent empathetic engagement delivered over the telephone by community-hired laypeople had a clinically meaningful effect on glycemic control for adults with low income and uncontrolled diabetes. Identifying how such a workforce might accompany and be coordinated with clinical care could accelerate achieving meaningful outcomes for patients and the health system.

## Notes

Supplement 1.
Trial Protocol and Statistical Analysis Plan

Supplement 2. **eTable 1.** Descriptive Statistics for All Measures for Women **eTable 2.** Descriptive Statistics for All Measures for Men

Supplement 3.
Data Sharing Statement
