# Supplementary material for: Glycemic Control With Layperson-Delivered Telephone Calls vs Usual Care for Patients With Diabetes: A Randomized Clinical Trial
Source: JAMA Netw Open. 2024 Dec 10;7(12):e2448809. doi: 10.1001/jamanetworkopen.2024.48809 (PMC11632544; doi:10.1001/jamanetworkopen.2024.48809)
Supplement: Supplement 1. — Trial Protocol and Statistical Analysis Plan [file jamanetwopen-e2448809-s001.pdf]

TEXAS
The University of Texas at Austin

FORM: IRB Proposal - Standard Submission	
NUMBER	VERSION DATE
HRP-UT901	04/29/2021

aGENERAL STUDY INFORMATION

Use for greater than minimal risk studies and minimal risk studies that fit into one or more expedited categories (see Section 5.3 of our Policies & Procedures for details regarding expedited research).

Do NOT submit this form if the study will qualify for exempt review, instead submit HRP-UT902 IRB Proposal – Exempt Submission Form found in the document Library.

If you are only using secondary data that will not be initially collected solely for this research project, use HRP-UT903 Template IRB Proposal Secondary Use form instead.

For studies following a multi-center or sponsor protocol, please use this guidance to assist in your completion of this form.

For questions regarding definitions, policies, or terms referenced below see the policies and procedures manual.

Please note, Word online does not support Word checkboxes. Please download the file and use your desktop version of Microsoft Word.

1 Review Type (Choose one)

*Click on the check box (or double click and type an "X" if using Google Docs) the **one** review type that applies.*

Please note: Expedited Review does not refer to the timeliness of the review of your protocol, but specific categories of research defined by ORHP. If you would like help determining which type of review is most appropriate for your study please contact the Office of Research Support and Compliance: https://research.utexas.edu/ors/about-ors/contact-us/.

a ☐ **Full Board Review – Greater than Minimal Risk Research**

b ☒ **Expedited Review – Minimal Risk Research**

2 Research Hypothesis

To input text, click in the light grey area below.

A program of empathetic and relationship-oriented telephone calls by lightly trained lay persons can support diabetes self-management behaviors and reduce Hemoglobin A1C in diabetic patients of a federally qualified health center (FQHC).

3 Study Background

To input text, click in the light grey area below.

Mental health issues affect an individual's ability to manage chronic health conditions such as diabetes (Chaoyang et al 2008; Schmitt et al 2021). In self-management programs for diabetes, when mental health is addressed it is usually secondary to more tactical self-management steps such as goal-setting and providing connection to resources. In this study we will assess a mental-health-first approach to supporting diabetes self-management through a program of empathetic telephone calls, delivered by lightly-trained lay people. The program builds on the success of a previously tested 4-week long empathetic telephone call program that found significant and sizeable reductions in depression, anxiety and general mental health (Kahlon et al 2021).

Prior work has shown that telephone-based diabetes education and support programs can significantly improve Hemoglobin A1C management, reducing levels in low income diverse communities. A program of 8 telephone calls over 12 months implemented in the Bronx, NY focusing primarily on medication adherence and secondarily on nutrition and exercise support achieved significant reductions of 0.8% relative to controls for those who began the program with A1C levels above 9% (Chamani et al 2015) A follow-up analysis showed that the intervention reduced hospitalization over the following three years (Tahbaei et al 2020).

In this study we will use a randomized controlled trial to assess the impact of a 6-month long intervention of empathetic relationship building engagement with emotional and motivational support delivered telephonically for patients with diabetes at a Federally Qualified Health Center (FQHC). Telephone callers, lay people with light training on how to be empathetic and engaging in calls, will support participants' journeys on their own goals related to nutrition, exercise, sleep and medication adherence. They will not provide diabetes specific information, instead they will support people walking more, sleeping better, eating better and taking their medications. The impact of the program will be assessed primarily on Hemoglobin A1C levels and secondarily on depression and mental health at the end of the 6-month intervention, and in the following 6 months. Results will be compared to patients in the control arm that receive usual care. Participants will be recruited from our partner, an FQHC, Lone Star Circle of Care (LSCC) and include patients with Hemoglobin A1C of greater than 8.0 as measured at any point in the 12 months preceding enrollment.

- Chaoyang Li, Earl S. Ford, Tara W. Strine, Ali H. Mokdad. Prevalence of Depression Among U.S. Adults With Diabetes Diabetes Care Jan 2008, 31 (1) 105-107; DOI: 10.2337/dc07-1154
- Kahlon MK, Aksan N, Aubrey R, et al. Effect of Layperson-Delivered, Empathy-Focused Program of Telephone Calls on Loneliness, Depression, and Anxiety Among Adults During the COVID-19

Pandemic: A Randomized Clinical Trial. JAMA Psychiatry. 2021;78(6):616–622. doi:10.1001/jamapsychiatry.2021.0113

- Schmitt, A., Bendig, E., Baumeister, H., Hermanns, N., & Kulzer, B. (2021). Associations of depression and diabetes distress with self-management behavior and glycemic control. *Health Psychology, 40*(2), 113–124. https://doi.org/10.1037/hea0001037
- Impact of a Telephonic Intervention to Improve Diabetes Control on Health Care Utilization and Cost for Adults in South Bronx, New York
- Bahman P. Tabaei, Renata E. Howland, Jeffrey S. Gonzalez, Shadi Chamany, Elizabeth A. Walker, Clyde B. Schechter, Winfred Y. Wu Impact of a Telephonic Intervention to Improve Diabetes Control on Health Care Utilization and Cost for Adults in South Bronx, New York. Diabetes Care Apr 2020, 43 (4) 743-750; DOI: 10.2337/dc19-0954

4 Design and Methodology

Provide information regarding study design or data collection methodologies. Details regarding protocol specific research procedures will be discussed in a later section.

To input text, click in the light grey area below.

Design: Randomized controlled trial.

Population: Adults with Hemoglobin A1C >8.0 measured at any point in the 12 months prior to enrollment, who have had at least one visit (Family medicine, behavioral health or other) with Lone Star Circle of Care within the past 12 months (in person or telehealth)

Randomization: Consented participants will be randomized to the intervention or control arm in a 1:1 ratio.

Blinding: Research staff involved in taking biomedical measurements will be blinded to participant status. Participants will complete surveys in person using a tablet or individual paper copy, thus research associates will remain blinded.

Time period: The study is divided into two parts.

- *Phase 1 (6 months)* is a randomized controlled trial testing the effect of telephone call-based empathetic engagement supported by caller-customized letters, together with small self-selected incentives (health tool) and caller-selected personal recognitions (small gifts selected based on the callers understanding of the participant), and low-literacy basic diabetes self-management information, against usual care. The primary outcome is Hemoglobin A1C (A1C) with repeated measures at baseline, 3 and 6 months.
- *Phase 2 (subsequent 6 months)* is the follow-up phase allowing us to *estimate* the 12 month longer term trajectory of A1C in Intervention and Control. To maximize the utility of the study to inform the most effective program design, participants in the control arm will receive the same opportunity to self-select health tool as the intervention arm received in Phase 1, but will make the choice during the 6

month survey collection. The material portion of the recognition/gifts, which (gift), will also be selected at the 6-month survey, but instead of being selected by the caller, the participant will select their own gift from the same set used by the callers in Phase 1. The control arm will also receive the low-literacy basic diabetes self-management information during Phase 2. All items will be provided to the control arm in Phase 2 on the similar schedule as intervention participants received them in Phase 1. As a result, we hope to *estimate* the impact of the small incentives and basic diabetes self-management information without the communication elements, telephone call-based empathetic engagement and customized newsletters, by comparing the results in the Intervention arm in Phase 1, with those in the Control arm in Phase 2.

Overview:

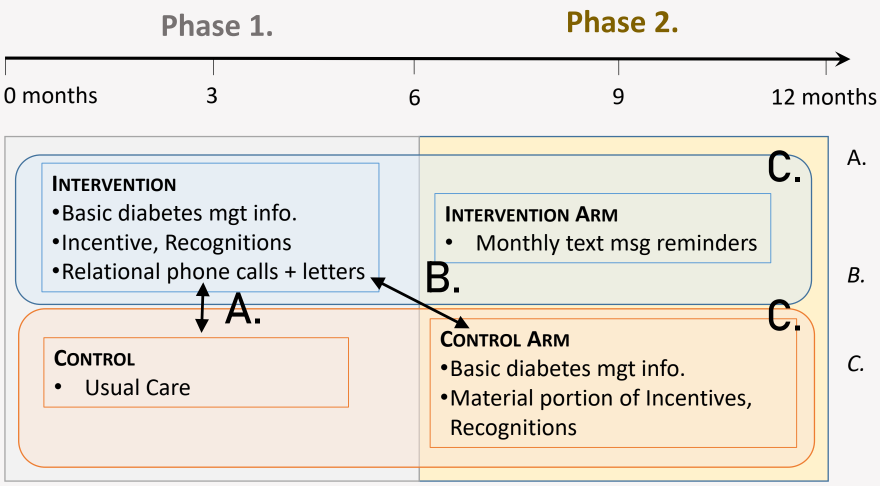

A. Assess effect of integration of small incentives + phone calls ("Program") against usual care: Randomized Controlled Trial in Phase 1
B. *Estimate* effect of phone calls alone: Intervention Phase 1 versus Control Phase 2.
C. *Estimate* Pre-Post changes in Intervention over 12 months (provides clues to sustainability) and control separately.

Intervention: Participants in the intervention arm receive -

- **Incentives & personal recognitions**
- Small Health-promoting Incentive:
  - Choice of a health-promoting tool offered by the caller in the first week of the program. A choice will be provided by the caller between a pedometer and a smart weigh scale of equivalent value. If neither of these options are applicable based on health or other limitations, back-up options will be offered to them (fitness resistant bands or exercise program for upper body fitness). The health-promoting tool will be mailed to participants by approximately the 5th week of the program.
- Personalized recognition of participant by caller ("Personal recognition/Gift"):
  - Callers will select, based on their understanding of the participant, 2 items from a larger pre-selected set of health-related items (related to cooking,

physical activity or sleep), each of equivalent ~$25.00 value. Callers will select and the program will mail items to the participants between months 1 and 5, to ensure the second item is received within 6 months (Phase 1). Callers will have a deadline to send the first item by the middle of the third month (Month 2.5) and by the middle of the 5th month (Month 4.5)

- Diabetes management information (print) geared to people with low-literacy (Diabetes management materials). This information will be mailed to intervention participants after randomization.
- **Communications to support and celebrate the participants learnings:**
  - Relationship-building empathetic telephone calls between lay caller and participant. Calls provide an opportunity for participants to discuss whatever they wish to share, and provide support for participants' goals for exercise, nutrition, sleep and medication adherence. In the course of engagement, if the caller feels like the participant can benefit from checking in with their regular clinic provider, they may suggest doing so. In addition, if a participant mentions a social need (such as transportation, food, housing), the caller may suggest some potential social resources for the participant to call. In addition, if the caller feels the participant could benefit from a pre-selected list of YouTube videos, that includes people talking about their life with diabetes, the caller may decide to share it with them. Calls occur 3X a week in first month and 1X a week in subsequent months for 6 months. Participants have agency in informing some aspects of how frequently they're called. After the first week they can adjust calls to a maximum of 5X a week or down to 2X a week (minimum of 2X/week for the first month). After the fourth week all calls shift to weekly. After the 8th week they can adjust calls to 2X every 4 weeks or stay on weekly schedule. Calls average 10 minutes per call or less. The participant may also receive communication via text message about their upcoming phone calls.
  - A letter sent twice in 6 months (Phase 1) to all participants in a callers panel of participants that reflect learnings from participants themselves. No identifiable information will be in these letters.
  - Automated Monthly text messages during months 7-12 (the follow-up phase) with pre-filled customized communications prepared by their caller as simple reminders of tips they discussed during the first 6 months.

Control: Participants in the control arm receive –

- **Material aspects of elements from the Intervention arm:**
- Diabetes management information for people with low literacy (print) at the start of phase 2 (after the 6 month assessment).
- Small health-promoting incentive:

- Choice of a health-promoting tool offered as one of the final questions of the 6 month assessment. A choice will be provided at the end of the 6 month surveys between a pedometer and a smart weigh scale of equivalent value. If neither of these options are applicable based on health or other limitations, back-up options will be offered to them (fitness resistant bands or exercise program for upper body fitness). The tool will be mailed within the 7th month.
- A choice 2 items from the same set of items used for the Intervention arm in Phase 1 related to healthier living, each <$25/-. This will be offered as one of the final questions of the 6 month assessment. The selected items will be mailed out to the participant in the 8th month and 10th month.

- Participants in the control arm do not receive the communications elements, telephone calls or letters, and otherwise continue with their normal standard of care.
- Participants in the control arm do not receive any text messaging reminders related to the program. To prevent loss to follow-up in the control arm, we will send research study reminder text messages about upcoming measurement appointments.

Measures: Primary outcome is Hemoglobin A1C at 6 months (repeated measures at 3 and 6, 9 and 12 months). The measurements will be taken at Lone Star clinic sites, Dell Medical School at The University of Texas at Austin, or other community-based sites, by a member of the research team. Secondary outcomes include Blood pressure through direct measurement and self-reported depression (PHQ-9), anxiety (GAD-7), general mental and physical health (SF-12) and self-reported healthcare utilization and will also be measured at Lone Star clinic sites, Dell Medical School at The University of Texas at Austin, or other community-based sites, by a member of the research team. In addition, process measures include measurements directly from the telephone call system: calls placed/attempted; calls connected (someone spoke); call duration. See below for table of all measures and method of collection.
Measure Collection: Biomedical measures will be directly collected in person at their clinic site, Dell Medical School at The University of Texas at Austin, or a community site that is convenient (A1C and Blood pressure). Patient-reported measures will be collected through self-report on a tablet (or paper copy if participant chooses) at the same time biomedical measures are collected. Electronic Health Record measures will be collected via a data agreement with Lone Star Circle of Care (LSCC).

Measure	Freq	Tool
**Program Effects**		
Hemoglobin A1C	Baseline/3 mths/6 mths/9 mths/12mths	Finger stick on portable device

Blood Pressure	Baseline/3 mths/6 mths/9 mths/12mths	Automatic blood pressure cuff
Depression	Baseline/3 mths/6 mths/9mths/12 mths	PHQ-9
Anxiety	Baseline/3 mths/6 mths/9 mths/12 mths	GAD-7
General Health	Baseline/3 mths/6 mths/9 mths/12 mths	SF-12
Healthcare Utilization (PCP & BH clinic, Hosp, ED visits)	Baseline/3 mths/6 mths/9 mths/12 mths	Customized (4 new questions added)
Healthcare Utilization (PCP visits, BH visits)	Data pulls for data collected during program	LSCC ElecHealthRecord
Medication Related to DM and HTN	Baseline/3 mths/6 mths/9 mths/12mths	Customized
Medication Adherence Survey	Baseline/3 mths/6 mths/9 mths12 mths	Med Adherence Report Scale (MARS 5)
Diabetes Self-Management	Baseline/3 mths/6 mths/9 mths/12 mths	Perceived Diabetes Self-Management Scale (PDSMS)
Diabetes Self Care	Baseline/3 mths/6 mths/9 mths/12 mths	Summary of Diabetes Self-Care Activities (SDSCA)
Diabetes Self Efficacy	Baseline/3 mths/6 mths/9 mths/12 mths	Stanford Self-Efficacy for Diabetes Scale
Diabetes Distress	Baseline/3 mths/6 mths/9 mths/12 mths	Diabetes Distress Screening Scale
Loneliness	Baseline/3 mths/6 mths/9 mths/12 mths	UCLA 3-item Loneliness Scale
Onset Date of Diabetes	Baseline	Customized
Social Needs Survey	Baseline/6 mths/12 mths	Adapted from PRAPARE
Social Support	Baseline	Lubben Social Network scale
Participant Demographics	Baseline	Customized
Household demographics	Baseline	Customized
Interest in Calling Others	6 mths	Customized
Program Satisfaction	6 mths (intervention arm only)	Customized
**Program Implementation**		
Call Occurrence	Continuous (daily; did it happen)	Twilio report
Call Connection	Continuous (daily; did the call connect to a person)	Twilio report
Call Duration	Continuous (daily); for every call placed, duration	Twilio report
Intracall dynamics	Continuous	Analysis of real-time audio streams

5 Data Analysis

Describe the data analysis plan, including any statistical procedures or power analysis.

To input text, click in the light grey area below.

Our primary outcome of interest is Hemoglobin A1C. However, the approach being used is modeled after a successful mental health intervention (Kahlon et al 2021) which showed depression can be improved with the type of empathy calls utilized in the current study.

To ensure that the effect of the empathy calls program on A1c can be assessed without confounding it with depressive mood in the first 6-month phase-I portion, we will rely on stratified randomization so that approximately 50% (N=75) of the individuals in both the control and experimental group have a PHQ9 ≥5 at

baseline and 50% of the individuals have PHQ9 <5 at baseline. With 150 individuals in each arm, three assessments for A1c, the primary outcome, and depression (PHQ9), secondary outcome, we have 80% statistical power with alpha = .05 to detect effects as small as f = .09, which is a small, standardized effect size, and have 90% power to detect effects as small as f = .10, also a small effect size, assuming only a moderate correlation for rank-order stability across time for each outcome of r = .30. This correlation is reasonable for A1c and is an underestimate for the typical correlation for PHQ9. Statistical power for the effect of interest increases as these correlations in the outcome measures goes up. Regarding correlations, we have 90% power to detect correlations as small as r = .26 and 80% power to detect correlations as small as r = .23 within each group (N = 150) at alpha = .05. These are fair to moderate effect sizes that bear on the power of any follow-up mediation analyses.

We will test differences in the trajectory of A1c over the course of six-months with three assessments (baseline, 3- and 6-months) for those who receive empathy calls to support diabetes self-management versus those who do not receive these support calls. We will rely on linear mixed effect regressions with random terms to model individual differences in both the intercept and change terms, accounting for the clustering effect of shared lightly trained callers. We will predict these trajectories using the following person-level covariates: grouping indicator, terms to capture diabetic treatment type and dose, change in PHQ9 from baseline to 6-month depression. The cross-level interaction of grouping indicator with time will constitute the effect of interest. In addition, if change in depression predicts the person-specific slopes for A1c and does so more strongly for those in the intervention than control group, we would have evidence that depression management affects A1c course. Similar models will be constructed for the secondary outcome of depression, PHQ9 across three waves of data collection, with frequency/duration of support calls across those waves modeled as a time-varying covariate. Person-level covariates will include grouping indicator, terms to capture change in A1c from baseline to 6-months. If change in A1c predicts person-specific slopes for depression and does so more strongly for those in the intervention than control, we would have preliminary evidence that improved control of A1c affects course of depression, an unlikely outcome. The outcome of these mixed effect regressions will permit a preliminary estimate of the mediational role of depression on A1c management in phase-I. We will estimate those indirect effects using bootstrapped standard errors using the MPlus modeling framework for longitudinal change from baseline to 6-months. Healthcare utilization outcomes both self-reported and obtained from LSCC will be harmonized into a composite score of utilization. Depending on the distribution of each aspect of utilization (ER visits, hospitalization, PCP visits), we will construct models to predict differences between the intervention and control arm while covarying change in A1c and depression from baseline to 12-months.

For phase-II of the study, we have the opportunity to estimate two effects of interest including: a) the effect of the incentive and materials provision (2-selected items and health tool) on A1c among controls in phase-II over a period of 6-months compared to its effects when such provisions are coupled with empathy calls among intervention participants in phase-I (a between group effect estimate), b) the effects of exposure periods (phase-I and phase-II) on A1c and depression from baseline through 12-months separately for the two groups. In the case of the intervention group, the phase-I to phase-II difference gives clues to sustainability and in the case of the control group, the phase-I to phase-II difference provides a within-person estimate of the effect of incentive provision

26 STUDY ELEMENT IDENTIFICATION

6 Study Elements

Click on the check box (or double click and type an "X" if using Google Docs) each procedure included in your study.

A full description of all study procedures should be provided in the Procedures (Details) section below and/or the applicable supplement form.

☒ Bio-specimens	☐ Biometrics	☐ Registry or Repository
☐ Focus Group	☐ Genetic Analysis	☐ Genomic Data Sharing
☐ International Research	☒ Interview/Survey	☐ MRI
☒ Protected Health Information	☒ Observation	☐ Radioactive Material/PET/Nuc. Med
☒ Record Review	☐ Sensors (Externally Placed)	☐ Sensors (Inserted)
☐ Video/Audio Recording	☐ X-Ray/CT	

27

7 Study Intervention

Click on the check box (or double click and type an "X" if using Google Docs) if you will implement any of the following interventions.

A full description of all study interventions should be provided in the Procedures (Details) section below and/or the applicable supplement form.

☒ Behavioral	☐ Device	☐ Drug/Biologic

28

29

8 Clinical Trial

Click on the following check box (or double click and type an "X" if using Google Docs) if the research meets the below definition of a clinical trial.

☒ **This study meets the definition of a clinical trial according to clinical trials.gov in that it involves one or more human subjects who are prospectively assigned to one or more interventions (which may include placebo or other control) to evaluate the effects of those interventions on health-related biomedical or behavioral outcomes.**

30

31

9 Additional Oversight

Click on the check box (or double click and type an "X" if using Google Docs) each activity that requires oversight from additional UT committees.

☐	Energy introduced to the subject (electrical, magnetic, light)	☐	Human embryonic, human induced pluripotent, or human totipotent stem cells; or human gametes or embryos	☐	Radiation exposure without direct clinical benefit

☒ **Biological Samples, Biohazards, Recombinant DNA, or Gene Transfer**

If biological samples are used and stored on UT campus IBC approval is needed.

a ☐ **Biological samples collected will not be stored on UT sites and another agency has responsibility for biospecimen safety.**

b **IBC Protocol Number**

To input text, click in the light grey area below.

IBC-2021-00220

10 Alternatives to Participation in This Study

To input text, click in the light grey area below.

The alternative to participation is usual care at Lone Star Circle of Care (LSCC), the FQHC where we will be recruiting participants.

STUDY PROCEDURE DESCRIPTION

11 Procedure Description

Describe all study procedures, including a step-by-step outline of what participants will be asked to do or how data will be used. Be sure to describe all of the following in detail, as applicable:

- Provide a description of all research procedures being performed and when they are performed, in sequential order.
- All research measures/tests that will be used and state if questions or measures are standardized or published (upload copies of all surveys, scripts and data collection forms)
- Secondary data or specimens that will be obtained, how they will be collected, and how they will be used
- Where each activity will take place, the duration of each, and who will perform each activity
- Include time commitment of participants

To input text, click in the light grey area below.

1. Recruitment
   a. Interest List
      i. A database will be created by Lone Star Circle of Care (LSCC) to include patients who meet inclusion and do not meet exclusion criteria. Messages will be sent out on the LSCC Care Message texting platform or emailed to identify interested participants from this database.
      ii. Lone Star Circle of Care (LSCC) staff will use flyers created by the research team to identify interested patients and a link to the recruitment flyer will be included in the CARE message text sent to LSCC patients when assessing interest in participating in the program.
   b. Consent
      i. Interested participants will be called by study staff to screen and share consent information. Consent will then be signed electronically, through DocuSign, or for those that do not complete this, the first time they come to a clinic site, Dell Medical School at The University of Texas at Austin, community-based site, or on rare occasion in people's homes, for baseline measurement, they will sign in person. No research activities will take place before informed consent is completed.
2. Measure collection
   a. General: Research associates/study staff will collect biomedical measures. Participants will fill out their own surveys on iPads/tablets at the measure collection site. Paper copies will be available for participants to complete upon request. Research associates will be blinded to the status of the participants.
   b. First measure collection
      i. After phone screening and consent, the participant will be invited to come the clinic site, Dell Medical School at The University of Texas at Austin, other community-based site, or on rare occasion in people's homes on weekends and/or in the evenings and given choices on when to schedule measurement collection. When they come to the site, they will get a chance to sign consent forms if they haven't done so electronically; then study staff will collect A1C and blood pressure measurements. After this, participants will use a tablet or paper copy to answer survey information. The survey

information will be saved directly into a RedCap database. The paper copy of the responses, if used, will be stored in a locked cabinet.

c. Subsequent measure collections (3, 6, 9 and 12 months)
    i. Similar to above, without informed consent process.

3. Randomization
    a. To ensure that the effect of the empathy calls program can be assessed without confounding it with depressive mood, we will rely on stratified randomization so that approximately 50% (N=75) of the individuals in both the control and experimental group have a PHQ9 > 5 at baseline and 50% of the individuals have PHQ9 < 5 at baseline. Randomization will occur after participant consent and collection of baseline measures.
4. Program Intervention
    a. Intervention arm
        i. After the participant has been randomized into the intervention arm, they will be allocated to a caller's panel by an unblinded program manager. Each caller has a panel of anywhere from 10-25 participants. Callers will place the first call within 1-5 days of receiving the name of a participant. Callers will use an app on their phone to be reminded of the schedule of calls and to initiate calls such that basic metrics can be collected (e.g., the call occurred, call duration, etc). Calls are not recorded.
        ii. First week of the program: Choice of a health-promoting tool offered by the caller in the first week of the program. A choice will be provided by the caller between a pedometer and a smart weigh scale of equivalent value. If neither of these options are applicable based on health or other limitations, back-up options will be offered to them (fitness resistant bands or exercise program for upper body fitness). The chosen health-promoting tool will be mailed out by the 5th week of the program. Low-literacy diabetes self-management information will also be mailed out after randomization occurs.
        iii. Month 2-5 of the program: Callers will select 2 items, one by Month 2.5 (7 weeks in), and one by Month 4.5 (18 weeks in), and the program will mail these items to participants as personal recognitions of the participants managing diabetes in their life. The items will each be of <$25.00 value, will come from a pre-selected list of items that are related to the main levers of diabetes self-management, nutrition, activity, sleep and med adherence. Callers will be allowed to add to the pre-selected list if they identify

something they think will be particularly well-received by the participant but isn't on the original list. That item will then become part of the super set of items that other callers can choose from. Examples of items include cookbooks, blender, children's cooking kit, spices, meal kits, gear to hold iPod and headphones while exercising, etc.

iv. Empathy Calling Program (Months 1-6): Relationship-building empathetic telephone calls between lay caller and participant. Calls provide an opportunity for participants to discuss whatever they wish to share, and provide support for participant's own goals on exercise, nutrition, sleep and medication adherence. In addition, if the caller feels the participant could benefit from a pre-selected list of YouTube videos, that includes people talking about their life with diabetes, the caller may decide to share it with them. Calls occur 3X a week in first month and 1X a week in subsequent months for 6 months. Participants have agency in informing some aspects of how frequently they're called. After the first week they can adjust calls to a maximum of 5X a week or down to 2X a week (minimum of 2X/week for the first month). After the fourth week all calls shift to weekly. After the 8th week they can adjust calls to 2X every 4 weeks or stay on weekly schedule. Calls average 10 minutes per call or less.

v. Customized letters to panel participants by each caller. The program will help transcribe and otherwise support via a template each caller describing learnings of their participants as they navigate life with diabetes, including trying to exercise or eat better. Anonymized learnings from participants in each caller's panel will be compiled in the letter, which will be physically mailed out 2 times between Months 2 and 5. Both letters will be uploaded to the IRB for approval prior to administration. The first letter will be uploaded to the IRB via modification first, and the second letter will be uploaded approximately a month after the first one to match with the mailing schedule.

vi. Follow-up Phase (Months 7-12): Automated Monthly text messages during months 7-12 (the follow-up phase) with pre-filled communications prepared by their caller as simple reminders of tips they discussed during the first 6 months.

vii. Time commitment for research measures = no more than 45 minutes for baseline and 30 minutes for every subsequent measure timepoint (3, 6, 9 and 12 months) Together with drive time to clinic,

Dell Medical School, or community center sites for measurements we estimate 1.5 hours for each measurement, occasionally at a maximum 2 hours, and our compensation is based on 2 hours per visit and 5 measurement visits.

viii. Time commitment for calls (estimated maximum) is:
- Week 1: 30 minutes or less
- Weeks 2-4: 50 minutes or less per week
- Months 2-6: 10 minutes or less per week

b. Control arm

i. Program Phase (Months 1-6): Control participants will receive their usual care during the first 6 months of the program.

ii. Follow-up Phase (Months 7-12): Through a survey at the end of the 6-month assessment, control participants are provided the same health tools and 'gift' options as the intervention arm was at the beginning of the program phase. They are provided a choice between a pedometer and a smart weigh scale (if neither of these options are applicable based on health or other limitations, back-up options will be offered to them (fitness resistant bands or exercise program for upper body fitness)); and they are provided a choice between the same set of items, each <\$25.00 and all related in some way to eating better, exercise, sleep or tools to help remember to take medications. The health tool and diabetes self-management information is delivered via mail on the same schedule as the intervention participants. The gifts selected by control participants will be mailed in Month 2 and Month 4.

iii. Time commitment for control measurements – same as for intervention, see above.

iv. Time commitment for control intervention – none.

35

36 SUBJECT POPULATION

12 Protected Subject Populations

Click on the check box (or double click and type an "X" if using Google Docs) each population, if they are specifically studied for this research.

☐ **Active military** ☐ **Children** ☐ **Decisionally impaired adults**

	personnel				
☐	Emancipated minors	☐	Fetuses	☐	Individuals with limited English proficiency
☐	Neonates	☐	Pregnant Woman	☐	Prisoners
☐	UT Students	☐	UT or Seton Staff/Employees		

37
38

13* Research Participant Information

Describe the research population.

**For multiple research populations (e.g., teachers, students, and parents), copy this section as necessary to describe your population.*

Participants are patients with diabetes who are seen at Lone Star Circle of Care, a Federally Qualified Health Center (FQHC) in the greater Austin region.

a Participant Group Name

To input text, click in the light grey area below.

Diabetic adults seen at Lone Star Circle of Care, a Federally Qualified Health Center

b Minimum Age

To input text, click in the light grey area below.

21

c Maximum Age

To input text, click in the light grey area below.

70

d Inclusion Criteria

To input text, click in the light grey area below.

Adults aged 21 to 70 years old
A1c >= ≥7.5 at baseline measurement and 8.0 at least one time in prior 12 months.
At least one visit with Lone Star Circle of Care within the past 12 months (in person or telehealth)
Must be willing to answer the PHQ-9 form in its entirety at baseline data collection due to stratified randomization design.

e Exclusion Criteria

To input text, click in the light grey area below.

A1c < 7.5 at baseline measurement. Refusal to answer the PHQ-9 form in its entirety at baseline data collection due to stratified randomization design. Moderate to severe cognitive impairment currently

pregnant, undergoing cancer treatment, diagnosis of end-stage renal disease or serious mental illness diagnosis, moderate to severe cognitive impairment, or receiving systemic treatment with prednisone or immunosuppressant therapy following an organ transplant.

f Additional Population Information

To input text, click in the light grey area below.

39

14 Total Sample Size

To input text, click in the light grey area below.

300

40

15 Sample size rationale

To input text, click in the light grey area below.

To ensure that the effect of the empathy calls program on A1c can be assessed without confounding it with depressive mood in the first 6-month phase-I portion, we will rely on stratified randomization so that approximately 50% (N=75) of the individuals in both the control and experimental group have a PHQ9 ≥5 at baseline and 50% of the individuals have PHQ9 <5 at baseline. With 150 individuals in each arm, three assessments for A1c, the primary outcome, and depression (PHQ9), secondary outcome, we have 80% statistical power with alpha = .05 to detect effects as small as f = .09, which is a small, standardized effect size, and have 90% power to detect effects as small as f = .10, also a small effect size, assuming only a moderate correlation for rank-order stability across time for each outcome of r = .30. This correlation is reasonable for A1c and is an underestimate for the typical correlation for PHQ9. Statistical power for the effect of interest increases as these correlations in the outcome measures goes up. Regarding correlations, we have 90% power to detect correlations as small as r = .26 and 80% power to detect correlations as small as r = .23 within each group (N = 150) at alpha = .05. These are fair to moderate effect sizes that bear on the power of any follow-up mediation analyses.

41 SCREENING AND RECRUITMENT

16 Identification and Screening

Click on the check box (or double click and type an "X" if using Google Docs) if true.

☒ **This study involves obtaining information or biospecimens for the purpose of screening, recruiting or determining eligibility of prospective subjects prior to informed consent by either:**

1. Oral or written communication with the prospective subject or LAR
2. By accessing records containing identifiable private information or stored identifiable biospecimens.

42

17 Identification and/or Screening Procedures

Describe the identification and/or screening procedures below.
To input text, click in the light grey area below.

i. A database will be created by Lone Star Circle of Care (LSCC) to include patients who meet inclusion and do not meet exclusion criteria.
ii. Messages will be sent out on the LSCC Care Message text platform or via email to identify interested participants from this registry.
iii. Lone Star Circle of Care (LSCC) staff will use flyers created by the team (and submitted with this IRB submission) to identify interested patients. A webpage link to the recruitment flyer will be included in the CARE message text sent to LSCC patients by LSCC staff.
iv. Research team members will call interested patients to explain the research and provide informed consent over the phone using DocuSign or in person at baseline visit.

43

18 Recruitment Overview

Click on the check box (or double click and type an "X" if using Google Docs) all recruitment methods utilized for this research.

☒	**E-mail**	☒	**Flyer**
☒	**In-Person**	☒	**Letter**
☐	**Social Media**	☐	**Research Pool**
☒	**Telephone/Text**	☐	**Snowball Sampling**
☐	**Web-post**	☐	**Word of Mouth**

44

19 Describe the recruitment process, including where recruitment will take place.

Describe the recruitment procedures below.
To input text, click in the light grey area below.

a. Interest List
   i. A participant qualifying list will be created by Lone Star Circle of Care (LSCC) to include patients who meet inclusion and do not meet exclusion criteria. Individuals with authorization will collect the initial PHI to create a list of patients that would like to be contacted by the research team. They will provide this list of names, e-mails, and phone numbers to the research team so we can call and provide

detailed information about the research and determine if patients would like to participate. Messages will be sent out on the LSCC Care Message text platform or email to identify interested participants from this database.

ii. Lone Star Circle of Care (LSCC) staff will use flyers created by the team (and submitted with this IRB submission) to identify interested patients.

iii. LSCC staff creating the interest list will be considered research team members and will be added to the IRB once they have been selected and trained (CITI).

b. Consent

i. Interested patients will be called by study staff to screen and share consent information. Consent will then be signed electronically, through DocuSign, or for those that do not complete this, the first time they come to their regular LSCC clinic site, Dell Medical School at The University of Texas at Austin, or community-based site, for baseline measurement, they will sign in person.

c. Measure collection – A1C measure

i. Research associates/study staff will collect HbA1C at in person visit after consent is signed. Individuals with HbA1C less than 7.5 will be considered a screen fail.

d. Measure collection – PHQ-9

i. After consent is signed and HbA1C is measured during the first in-person visit, research associates/study staff will give a tablet or paper copy to the participant for them to complete the questionnaires. One of the first few questionnaires is the PHQ-9, which will have all questions marked as required, so that the participant cannot move ahead in the surveys unless each question is answered. This approach is needed to ensure adequate and fair stratified randomization. Participants that flag to the research associates/study staff that they do not want to complete the PHQ-9 questionnaire will be informed that they will not be able to join the study.

45

46 OBTAINING INFORMED CONSENT

20 Consent Overview

Click on the check box (or double click and type an "X" if using Google Docs) all applicable items.

☒ **Obtaining Written Informed Consent**

☐ **Requesting a Waiver of Documentation of Informed Consent**

☐ **Requesting a Waiver of Informed Consent**

☐ **Requesting an Alteration of the Required Elements of Informed Consent**

☐ **Obtaining Child Assent**

☐ **Obtain Consent Using a Short Form with a Witness**

47

21 Consent and Assent Processes

Provide a detailed description of the consent process including who will obtain consent, where, and when consent will occur in such a manner that participants have sufficient time for adequate consideration.

To input text, click in the light grey area below.

i. Interested patients will be called by study staff to screen and share consent information. Consent will then be signed electronically, through DocuSign, or for those that do not complete this, the first time they come to a clinic site, Dell Medical School, other community-based site, or on rare occasion at people's homes, for baseline measurement, they will sign in person. Study staff/research associates will share detailed information about the consent and be present in person to get signatures and subsequently, baseline measures at the first timepoint.

48

22 Consent and Translation

Click on the check box (or double click and type an "X" if using Google Docs) to indicate that consent will be translated.

☒ **The study population will likely include participants whose limited English speaking status requires translation of the consent form.**

Translation Process

Click on the check box (or double click and type an "X" if using Google Docs) that best describes the translation process, either 21 or 22.

23 ☐ **The consent documents will be translated by a certified translator.**

24 ☒ **A non-certified translator will translate the consent documents.**

i Describe the translator's qualifications

To input text, click in the light grey area below.

Two English fluent and native Spanish speakers within research personnel. One will translate and the other will confirm translation.

ii ☒ **Another individual will confirm that the translation is accurate and appropriate.**

49

Waiver of Documentation of Informed Consent

To approve a waiver of documentation of informed consent, one of the following options below must be justified by the researcher.

Only complete the sections below if requesting a waiver of documentation of informed consent. *If not requesting a waiver of documentation of consent, skip to 27.*

Please choose one waiver option and provide additional information as prompted. The Office of Research Support and Compliance recommends using Waiver Option 2 in most cases.

25 Waiver Option 1

Provide confirmation for the following criteria and follow the additional instructions.

Additional Instructions:

1. Include this choice in the informed consent form.

2. Articulate the destruction process for signed consent forms in the privacy and confidentiality section.

Click on the check box (or double click and type an "X" if using Google Docs).

a ☐ **The only record linking the subject and the research would be the consent document.**

b ☐ **The principal risk would be potential harm resulting from a breach of confidentiality.**

c ☐ **Each subject will be asked whether the subject wants documentation linking the subject with the research, and the subject's wishes will govern.**

26 Waiver Option 2

Provide confirmation for the following criteria and follow the additional instructions.

Click on the check box (or double click and type an "X" if using Google Docs).

a ☐ **The study is minimal risk.**

b ☐ **Written consent would not be required outside the research context.**

27 Waiver Option 3

Provide confirmation for the following criteria and provide additional information as requested.

Click on the check box (or double click and type an "X" if using Google Docs).

a ☐ **The subjects or legally authorized representatives are members of a distinct cultural group or community in which signing forms is not the norm**

b **Describe the cultural group or community.**

To input text, click in the light grey area below

c ☐ **The research presents no more than minimal risk of harm to subjects.**

d ☐ **There is an appropriate alternative mechanism for documenting that informed consent was obtained.**

e **Describe mechanism for documenting that informed consent was obtained**

To input text, click in the light grey area below

50

Waiver or Alteration of Informed Consent

To approve a waiver or alteration of informed consent all of the following criteria below must be justified by the researcher.

Only complete the sections below if requesting a waiver of informed consent. *If not requesting a waiver or alteration of consent, skip to 31.*

28 The research involves no more than minimal risk to the subjects.

To input text, click in the light grey area below

29 **The waiver or alteration will not adversely affect the rights and welfare of the subjects.**

To input text, click in the light grey area below

30 **The research could not practicably be carried out without the waiver or alteration (it is impracticable to perform the research if obtaining informed consent is required and not just impracticable to obtain consent).**

To input text, click in the light grey area below

31 **If the research involves using identifiable private information or identifiable biospecimens, the research could not practicably be carried out without using such information or biospecimens in an identifiable format.**

To input text, click in the light grey area below.

51

52

Deception and Debriefing

Only complete the sections below if requesting an alteration of informed consent that involves deceiving research participants. *If this study does not involve deception, skip to 35.*

See IRB Policies and Procedures Section 15 for a description of deception.

Click on the check box (or double click and type an "X" if using Google Docs).

32 ☐ **It is appropriate to provide additional pertinent information to the subject after research activities are complete (e.g., the researcher needed to deceive to subject to the nature of the study).**

33 ☐ **Research participants will have the opportunity to withdrawal their data during the debriefing.**

34 **Describe the nature of deception and why it is necessary to conduct the research.**

To input text, click in the light grey area below.

35 **Describe debriefing procedures.**

To input text, click in the light grey area below.

53

BENEFITS

36

Benefits to Society

Describe the scientific and societal benefit(s) below.

To input text, click in the light grey area below.

We hypothesize that providing empathetic and relationship-oriented telephone calls by lightly trained lay persons can support diabetes self-management behaviors and reduce Hemoglobin A1c.   By measuring the impact of such a lay person driven program on Hemoglobin A1C and mental health we plan to build a case for the health system to pay for better outcomes through such programs and influence the development of a novel workforce.

54

Benefits to Participants

Click on the applicable check box (or double click and type an "X" if using Google Docs).

37 ☐ **There is no anticipated direct benefit to participants.**

38 ☒ **There are anticipated benefits to participants.**

39 **If applicable, describe the potential direct benefits to participants.**

To input text, click in the light grey area below.

- Participants in the intervention arm will receive phone calls that may make them feel better by having someone listen and empathize with them.

RISKS

40 **Describe the risks associated with each activity in this research**

To input text, click in the light grey area below.

- Finger stick blood sample for HbA1c blood testing: The risks of obtaining a blood sample are minimal, similar to any time blood is collected and include pain, bleeding, bruising, infection, and skin irritations (from cleaning agents used to sterilize the skin or bandages).
- Blood Pressure: Participants may experience mild discomfort in their arm when the cuff is inflated.
- Loss of confidentiality: There is a potential for accidental release of confidential information. Procedures are in place to minimize this risk.

41 **Describe how each risk is mitigated/minimized.**

To input text, click in the light grey area below.

- Study team members collecting BP and blood samples will be trained on proper technique to maintain safety and minimize discomfort
- Participant study numbers and a secure REDCap database will be used to protect patient confidentiality.

Data Safety Monitoring

For additional information regarding data safety monitoring boards and data safety monitoring plans, please see Section 21 of our Policies and Procedures.

Click on the check box (or double click and type an "X" if using Google Docs).

42 ☒ **This study is minimal risk and does not require a Data Safety Monitoring Plan (DSMP) or a Data Safety Monitoring Board (DMSB).**

43 ☐ **This study does not have a Data Safety Monitoring Board, but researchers have an internal plan/policy to monitor for safety.**

Complete Data Safety Monitoring Details (44-51).

44 ☐ **This study has a Data Safety Monitoring Board (DSMB).**

Complete Data Safety Monitoring Details (44-51) or upload this study's Data Safety Monitoring Board's charter.

59

Data Safety Monitoring (Details)

45 **How is safety information collected?**

To input text, click in the light grey area below.

46 **When will safety data collection start (for each participant or for the whole study, as applicable)?**

To input text, click in the light grey area below.

47 **How frequently will safety data be collected?**

To input text, click in the light grey area below.

48 **Who will review the data for safety?**

To input text, click in the light grey area below.

49 **How frequently will data be monitored for safety concerns?**

To input text, click in the light grey area below.

50 **What data will be reviewed?**

To input text, click in the light grey area below.

51 **State the frequency or periodicity of the review of cumulative data?**

To input text, click in the light grey area below.

52 **State any conditions that would trigger an immediate suspension of the research.**

To input text, click in the light grey area below.

60

Early Withdrawal

Only complete this section if there are planned conditions under which a participant will be withdrawn from the study. *If not applicable, skip to 56.*

Include this information in your consent form.

53 **List the criteria for withdrawing individual participants from the study (e.g., safety or toxicity concerns, emotional distress, inability to comply with the protocol, or requirements from study sponsor).**

To input text, click in the light grey area below.

Participants may be withdrawn from the study if they verbally abusive or inappropriate with callers.

54 **Describe any necessary procedures for ensuring the safety of a participant who has withdrawn early.**

To input text, click in the light grey area below.

There are no procedures in place to ensure the safety of a participant that is withdrawn for abusive or inappropriate behavior toward callers. This is a minimal risk study and being withdrawn poses no risk to the participant.

55 **Describe any pre-specified criteria for stopping or changing the study protocol due to safety concerns.**

To input text, click in the light grey area below.

None

REQUIRED DISCLOSURES

Required Consent Disclosures

Identify each element below that may require additional information to be disclosed in the consent form.

Click on the check box (or double click and type an "X" if using Google Docs).

56 ☒ **It is reasonable that researchers could discover or suspect child or elder abuse.**

57 ☐ **It is reasonable that researchers could learn of an incident that could require reporting under Title IX.**

58 ☒ **It is reasonable that researchers could discover incidental findings or other information of medical interest about a participant's previously unknown condition.**

59 **Articulate methods for addressing and reporting incidental findings, if applicable.**

To input text, click in the light grey area below.

If during the study, we learn about child or elder abuse or neglect, or that someone is a clear, serious, and direct harm to self or others, we may report the information to the appropriate authorities, including the police, the Texas Department of Family and Protective Services, Lone Star Circle of Care and/or an emergency medical facility.

In the event a participant includes a response of 1-3 on the PHQ-9 question #9, the following protocol will be activated:

1. REDCap real-time notification via email to assigned research staff person while participant completes remaining surveys
2. Research team alerts Dr. M. Renee Valdez, Director of Psychiatry and BH at LSCC, immediately and normal LSCC procedures will occur to escalate issue to offer resources (direct phone call by BH provider)

PRIVACY AND CONFIDENTIALITY

60 Privacy

Describe how you will protect the identity and privacy of study participants during each phase of research. Privacy focuses on the individual participants rather than data. In this section, researchers should focus on issues such as where research activities take place and how participant involvement is protected from non-participants.

Describe methods to ensure participants' privacy during identification, recruitment, screening, the consent process, the conduct of the study, and dissemination of data.

To input text, click in the light grey area below.

Participant privacy will be maintained by conducting research visits in a private room or if in a large space, by providing adequate distance to ensure privacy. Surveys will be completed by participants using tablets further ensuring privacy. If participant chooses to complete surveys with a hard copy, it will be stored in a locked cabinet before and after measurement collection. All intervention procedures (phone calls) will be conducted using a phone number provided by the participant.

Confidentiality and Data Security Plan

Click on the check box (or double click and type an "X" if using Google Docs) that best describes the confidentiality and data security plan and provide additional details regarding how you will protect the confidentiality of data or address confidentiality concerns.

61 ☐ Identifiers will be coded to protect confidentiality.

61a If true, state how data is coded and where identifiers are stored.

To input text, click in the light grey area below.

62 ☒ Identifiable data will be destroyed.

62a If true, describe destruction plan and timeline

To input text, click in the light grey area below.

Identifiers will be destroyed at the end of the study after all data has been analyzed. All data will be stored in the Dell Med REDCap database. This secure, HIPAA compliant database can only be accessed with password and dual authentication. Hard copies of the measurements will be available to participants upon request. If participant chooses to complete measurements with a paper copy, responses will be entered in the REDCap database, and the paper copy will be stored in a locked cabinet until it is destroyed with the other data (after it has been analyzed).

63 ☐ **Identifiable data will not be destroyed.**

63a If true, provide rationale for retaining identifiable data indefinitely.

To input text, click in the light grey area below.

67

68

64 Data Access

Click on the check box (or double click and type an "X" if using Google Docs) for each group of individuals that will have access to study data.

If you plan on creating a repository, complete the repository form as well.

☒ **Study Team Members**

☒ **External Collaborators**

☐ **Data coordinating center**

☐ **Sponsor**

☒ **Future Sharing with other researchers**

☐ **Others**

Describe below. To input text, click in the light grey area below.

65 Describe data sharing plan for each group checked above and state whether researchers plan on sharing identifiable, coded, or de-identified data

To input text, click in the light grey area below.

No identifiable research data will be shared outside of the research team. Aggregate data will be shared with LSCC and presented in public.

We may share data with other researchers for future research studies that may be similar to this study or may be very different. The data shared with other researchers will not include information that can directly identify the participant

69

Certificate of Confidentiality

Click on the check box (or double click and type an "X" if using Google Docs) to identify each element below that may require additional information to be disclosed in the consent form.

If a Certificate of Confidentiality is not applicable for this study, skip to 68.

66 ☐ **The study requires a Certificate of Confidentiality.**

67 ☐ **NIH has issued a Certificate of Confidentiality for this study.**

68 ☐ **A Certificate of Confidentiality has not been obtained, but there are plans to apply for one.**

70

COMPENSATION AND COSTS

Compensation

Click on the check box (or double click and type an "X" if using Google Docs).

69 ☒ **Subjects receive compensation.**

70 ☐ **Subject will not receive compensation.**

Skip to question 74 if subjects will not receive compensation.

71 **Total Amount of Compensation**

To input text, click in the light grey area below.

Participants will be paid $50.00 for each measurement visit for a maximum of $250.00 for 5 visits.

72 **Type of Compensation**

Click on the check box (or double click and type an "X" if using Google Docs) for each form of compensation that will be provided.

☐ Cash	☐ Check	☒ Gift Card
☐ Course Credit	☐ ClinCard	☒ Tango Card

☐ **Other**

Describe, To input text, click in the light grey area below.

71

73 **Proration Schedule**

To input text, click in the light grey area below.

Participants will be paid $50.00 for each measurement visit for a maximum of $250.00 for 5 visits.

72

74 ☒ **Amount of compensation and its form is reasonable for this population for the activities requested of them.**

73

75 **Costs**

Click on the check box (or double click and type an "X" if using Google Docs) each applicable item regarding

costs.

☐ **Participants will have no costs associated with this study**

☐ **Standard of care procedures contributing to study data**

☐ **Research procedures not associated with standard of care**

☐ **Administration of drugs / devices**

☐ **Study drugs or devices**

☒ **Transportation and parking**

76 **Describe all costs below.**

To input text, click in the light grey area below.

There is a small cost to travel to the study visit sites. This will vary from one participant to the next, but will be negligible for most.

74
