# Supplementary material for: Glycemic Control With Layperson-Delivered Telephone Calls vs Usual Care for Patients With Diabetes: A Randomized Clinical Trial
Source: JAMA Netw Open. 2024 Dec 10;7(12):e2448809. doi: 10.1001/jamanetworkopen.2024.48809 (PMC11632544; doi:10.1001/jamanetworkopen.2024.48809)
Supplement: Supplement 2. — eTable 1. Descriptive Statistics for All Measures for Women eTable 2. Descriptive Statistics for All Measures for Men [file jamanetwopen-e2448809-s002.pdf]

Supplementary Online Content

Kahlon MK, Aksan NS, Aubrey R, et al. Glycemic control with layperson-delivered telephone calls vs usual care for patients with diabetes: a randomized clinical trial. *JAMA Netw Open.* 2024;7(12):e2448809. doi:10.1001/jamanetworkopen.2024.48809

eTable 1. Descriptive Statistics for All Measures for Women

eTable 2. Descriptive Statistics for All Measures for Men

This supplementary material has been provided by the authors to give readers additional information about their work.

© 2024 Kahlon MK et al. *JAMA Network Open.*

eTable 1. Descriptive Statistics for All Measures for Women

Outcome	Groups	@B	@3mos	@6mos
		M (SD) n	M (SD) n	M (SD) n
A1c	UC-PHQ9<5	9.72 (1.46) 45	9.63 (1.78) 41	9.42 (1.90) 38
	INT-PHQ9<5	9.85 (1.97) 45	9.93 (2.23) 37	9.51 (2.19) 37
	UC-PHQ9>=5	10.15 (1.77) 40	9.77 (2.21) 34	9.92 (2.62) 31
	INT-PHQ9>=5	9.81 (1.60) 33	9.13 (1.92) 27	8.66 (1.74) 28
PHQ9	UC-PHQ9<5	1.96 (1.49) 45	2.08 (2.42) 38	1.63 (1.99) 35
	INT-PHQ9<5	1.02 (1.27) 45	1.52 (1.68) 33	1.24 (1.48) 38
	UC-PHQ9>=5	10.43 (5.18) 40	8.66 (6.36) 32	8.42 (5.57) 31
	INT-PHQ9>=5	10.58 (5.34) 33	8.58 (5.74) 26	7.39 (4.67) 28
GAD7	UC-PHQ9<5	2.80 (2.94) 45	2.59 (2.61) 41	2.13 (2.89) 38
	INT-PHQ9<5	1.62 (2.04) 45	2.11 (2.88) 37	1.16 (1.79) 38
	UC-PHQ9>=5	8.50 (4.93) 40	9.21 (7.09) 34	7.06 (5.11) 31
	INT-PHQ9>=5	9.24 (6.05) 33	9.00 (6.11) 27	8.68 (5.69) 28
MH	UC-PHQ9<5	0.10 (0.09) 45	0.10 (0.08) 38	0.08 (0.08) 35
	INT-PHQ9<5	0.06 (0.06) 45	0.08 (0.09) 33	0.05 (0.06) 38
	UC-PHQ9>=5	0.40 (0.18) 40	0.37 (0.25) 32	0.32 (0.21) 31
	INT-PHQ9>=5	0.42 (0.22) 33	0.38 (0.24) 26	0.34 (0.21) 28
PDSMS	UC-PHQ9<5	23.29 (5.83) 45	25.95 (5.65) 41	26.11 (5.89) 38
	INT-PHQ9<5	25.02 (6.29) 45	25.54 (6.76) 37	27.16 (4.90) 38
	UC-PHQ9>=5	21.82 (4.69) 40	22.65 (5.87) 34	23.39 (6.29) 31
	INT-PHQ9>=5	20.12 (5.15) 33	23.70 (4.87) 27	24.82 (6.54) 28
DDSS	UC-PHQ9<5	6.76 (2.76) 45	7.54 (2.78) 41	7.95 (2.71) 38
	INT-PHQ9<5	7.42 (3.12) 45	8.57 (2.51) 37	8.71 (2.50) 38
	UC-PHQ9>=5	5.78 (2.60) 40	5.50 (3.02) 34	6.48 (3.19) 31
	INT-PHQ9>=5	5.00 (2.62) 33	5.52 (2.83) 27	6.68 (2.89) 28
SEDS	UC-PHQ9<5	45.91 (17.35) 45	48.12 (16.82) 41	55.26 (18.37) 38
	INT-PHQ9<5	53.84 (16.17) 45	55.30 (19.23) 37	58.58 (19.12) 38
	UC-PHQ9>=5	34.33 (15.71) 40	42.09 (15.42) 34	39.71 (19.23) 31
	INT-PHQ9>=5	39.00 (12.34) 33	46.81 (15.10) 27	47.04 (17.38) 28
MARS	UC-PHQ9<5	21.33 (3.57) 45	21.59 (4.01) 41	22.03 (3.97) 3
	INT-PHQ9<5	22.27 (3.49) 45	21.62 (3.51) 37	21.47 (4.35) 38
	UC-PHQ9>=5	18.93 (4.67) 40	19.44 (4.63) 34	19.16 (4.44) 31
	INT-PHQ9>=5	18.03 (3.53) 33	19.33 (4.23) 27	20.25 (3.04) 28
DMEH	UC-PHQ9<5	0.64 (0.12) 45	0.69 (0.13) 41	0.72 (0.13) 38
	INT-PHQ9<5	0.70 (0.13) 45	0.73 (0.15) 37	0.75 (0.14) 38
	UC-PHQ9>=5	0.55 (0.11) 40	0.58 (0.16) 34	0.60 (0.17) 31
	INT-PHQ9>=5	0.53 (0.10) 33	0.60 (0.11) 27	0.64 (0.13) 28

Abbrv. @B = at baseline; mos=months; A1c= Hemoglobin A1c; DDSS= Diabetes Distress Screening Scale; GAD7= Generalized Anxiety Disorder; MARS = Medication Adherence Report Scale; PDSMS = Perceived Diabetes Self-

© 2024 Kahlon MK et al. *JAMA Network Open.*

Management Scale; SEDS = Stanford Self-Efficacy for Diabetes Scale; PHQ9=Patient Health Questionnaire-9; MH = Mental Health composite score; DMEH=Diabetes related emotion health composite score

Notes. High scores in PDSMS, DDSS, MARS, SEDS reflect better DM management, less distress, better adherence and higher efficacy; and high values in their composite score DMEH, reflect better diabetes related emotional health. High scores in PHQ9 and GAD7 and their composite MH reflect greater symptoms of depression/ anxiety.

© 2024 Kahlon MK et al. *JAMA Network Open.*

eTable 2. Descriptive Statistics for All Measures for Men

Outcome	Groups	@B	@3mos	@6mos
		M (SD) n	M (SD) n	M (SD) n
A1c	UC-PHQ9<5	9.58 (1.72) 35	9.37 (1.44) 19	9.45 (1.92) 21
	INT-PHQ9<5	10.01 (1.76) 35	9.84 (2.13) 31	9.63 (2.09) 32
	UC-PHQ9>=5	10.01 (1.52) 10	10.87 (2.31) 7	12.04 (3.02) 5
	INT-PHQ9>=5	10.81 (2.18) 15	10.00 (2.09) 14	9.60 (1.73) 10
PHQ9	UC-PHQ9<5	1.31 (1.49) 36	1.00 (1.41) 18	1.70 (2.08) 20
	INT-PHQ9<5	1.40 (1.33) 35	1.27 (1.82) 30	2.16 (4.98) 31
	UC-PHQ9>=5	12.90 (5.24) 10	9.50 (4.76) 6	11.00 (3.08) 5
	INT-PHQ9>=5	8.40 (4.61) 15	6.93 (5.89) 14	8.30 (5.98) 10
GAD7	UC-PHQ9<5	2.53 (4.40) 36	1.37 (3.32) 19	2.33 (3.20) 21
	INT-PHQ9<5	1.89 (2.93) 35	1.74 (2.56) 31	1.34 (2.10) 32
	UC-PHQ9>=5	11.20 (6.14) 10	8.00 (5.03) 7	9.20 (3.83) 5
	INT-PHQ9>=5	7.80 (5.07) 15	6.43 (6.30) 14	9.30 (5.93) 10
MH	UC-PHQ9<5	0.08 (0.12) 36	0.05 (0.10) 18	0.09 (0.11) 20
	INT-PHQ9<5	0.07 (0.08) 35	0.07 (0.08) 30	0.07 (0.11) 31
	UC-PHQ9>=5	0.51 (0.22) 10	0.39 (0.19) 6	0.42 (0.09) 5
	INT-PHQ9>=5	0.34 (0.20) 15	0.28 (0.26) 14	0.38 (0.23) 10
PDSMS	UC-PHQ9<5	24.94 (5.99) 36	28.16 (6.73) 19	27.38 (5.77) 21
	INT-PHQ9<5	25.09 (5.42) 35	26.77 (6.17) 31	28.62 (6.08) 32
	UC-PHQ9>=5	18.50 (5.64) 10	20.00 (6.86) 7	19.40 (4.39) 5
	INT-PHQ9>=5	22.47 (7.40) 15	24.36 (8.54) 14	25.40 (7.86) 10
DDSS	UC-PHQ9<5	8.14 (2.86) 36	9.05 (2.76) 19	8.86 (3.04) 21
	INT-PHQ9<5	8.03 (2.77) 35	8.39 (2.78) 31	8.81 (2.63) 32
	UC-PHQ9>=5	5.30 (3.83) 10	4.71 (2.69) 7	4.60 (2.30) 5
	INT-PHQ9>=5	5.93 (3.20) 15	7.21 (3.47) 14	7.40 (2.88) 10
SEDS	UC-PHQ9<5	53.86 (20.09) 36	59.63 (17.66) 19	50.43 (20.37) 21
	INT-PHQ9<5	49.80 (22.34) 35	51.19 (21.56) 31	55.38 (19.26) 32
	UC-PHQ9>=5	37.70 (14.86) 10	41.29 (19.09) 7	38.20 (4.87) 5
	INT-PHQ9>=5	38.67 (15.55) 15	49.57 (18.29) 14	50.60 (19.22) 10
MARS	UC-PHQ9<5	21.58 (3.81) 36	23.05 (2.34) 19	20.33 (5.68) 21
	INT-PHQ9<5	20.71 (3.13) 35	20.97 (3.25) 31	22.06 (2.77) 32
	UC-PHQ9>=5	20.10 (4.70) 10	20.43 (3.87) 7	19.00 (3.61) 5
	INT-PHQ9>=5	17.47 (4.96) 15	19.50 (5.32) 14	17.80 (5.07) 10
DMEH	UC-PHQ9<5	0.71 (0.14) 36	0.78 (0.12) 19	0.72 (0.15) 21
	INT-PHQ9<5	0.69 (0.15) 35	0.71 (0.13) 31	0.76 (0.12) 32
	UC-PHQ9>=5	0.54 (0.15) 10	0.56 (0.15) 7	0.53 (0.07) 5
	INT-PHQ9>=5	0.56 (0.15) 15	0.65 (0.21) 14	0.65 (0.20) 10

Abbrv. @B = at baseline; mos=months; A1c= Hemoglobin A1c; DDSS= Diabetes Distress Screening Scale; GAD7= Generalized Anxiety Disorder; MARS = Medication Adherence Report Scale; PDSMS = Perceived Diabetes Self-

© 2024 Kahlon MK et al. *JAMA Network Open.*

Management Scale; SEDS = Stanford Self-Efficacy for Diabetes Scale; PHQ9=Patient Health Questionnaire-9; MH = Mental Health composite score; DMEH=Diabetes related emotional health composite score

Notes. High scores in PDSMS, DDSS, MARS, SEDS reflect better DM management, less distress, better adherence and higher efficacy; and high values in their composite score DMEH, reflect better diabetes related emotional health. High scores in PHQ9 and GAD7 and their composite MH reflect greater symptoms of depression/ anxiety.

© 2024 Kahlon MK et al. *JAMA Network Open.*
