# Supplementary material for: Glycemic Control With Layperson-Delivered Telephone Calls vs Usual Care for Patients With Diabetes: A Randomized Clinical Trial
Source: JAMA Netw Open. 2024 Dec 10;7(12):e2448809. doi: 10.1001/jamanetworkopen.2024.48809 (PMC11632544; doi:10.1001/jamanetworkopen.2024.48809)
Supplement: Supplement 3. — Data Sharing Statement [file jamanetwopen-e2448809-s003.pdf]

Data Sharing Statement

Kahlon. Glycemic Control With Layperson-Delivered Telephone Calls vs Usual Care for Patients With Diabetes. *JAMA Netw Open*. Published December 10, 2024. doi:10.1001/jamanetworkopen.2024.48809

Data
Additional Information: Clinicaltrials.gov #NCT05173675
Data available: Yes
Data types: Deidentified participant data, Data dictionary
How to access data: Will work with university to determine approach.
When available: beginning date: 12-15-2024

Supporting Documents
Document types: Informed consent form
How to access documents: Available on request (or uploaded to journal)
When available: With publication

Additional Information
Who can access the data: researchers whose proposed use of the data has been approved
Types of analyses: for scientific purposes.
Mechanisms of data availability: with a signed data access agreement
